# Micro-Household Human Capital Investment Decisions and a Simulation Study from the Intergenerational Conflict Perspective

**DOI:** 10.3390/ijerph20031696

**Published:** 2023-01-17

**Authors:** Qiling Lu, Jing Hua

**Affiliations:** College of Economics and Management, Ningxia University, Yinchuan 750021, China

**Keywords:** population aging, family educational investment, multi-agent simulation, childcare burden, crowding-out effect

## Abstract

Education is highly valued in Asian families. However, as family members age, competition for intra-family resources affects children’s actualization in the family, which impacts the family’s future capital. However, most existing studies have interpreted the family’s intergenerational conflicts in terms of care services for older adults, and few have analyzed and simulated intra-family competition based on the intergenerational conflict. This study introduces a multi-agent simulation approach to observe micro-households’ educational investment choices under the dual pressures of retirement and childcare. This measure captures households’ investment choices and provides a decision basis for given households. Using data from the China Family Panel Study for 2014, 2016, and 2018, we explore the impact of these dual pressures on household educational expenditures and their differences across urban and rural areas, household aging, and income samples. We also simulated the micro-households’ investment choices under these dual pressures to observe that these pressures reduce investments in educational human capital in these “sandwich-like” households. The simulation results suggest that households with high childcare stress invest more in education than those with a high retirement burden. Moreover, income growth can mitigate the dual stress “crowding-out” effect on education, which is most pronounced in low-income, high childcare-stress households.

## 1. Introduction

As countries pursue socioeconomic development, they have also begun to differentiate in their implementation of population-based policies. For example, many developed countries have discovered that low fertility and rapid aging have actually affected socioeconomic development [1]. These countries have gradually begun to shift from their previous one-sided focus on controlling absolute population numbers to their current strategy of jointly optimizing their population’s structure and quality [2]. In less than 30 years, China’s implementation of a family planning policy has moved its population into a modern pattern of population growth, with low birth and death rates, a low natural growth rate, and an accelerating pace of aging [3]. Its seventh census indicates that the population aged 60 and older accounted for 18.7% of the population in 2020, which is an increase of 5.44 percentage points compared with the data from the sixth census; the population aged 65 and older accounted for 13.5% of the total population size, or an increase of 4.63 percentage points compared with the sixth census.

In response to its growing problem of aging, in 2019, China’s central government proposed a strategic goal to actively address population aging, deploying a series of specific tasks in five areas. Simultaneously, China’s three-child policy officially began in 2021 to strengthen the nation’s population-development strategy and promote its long-term balanced development. The 2022 20th Party Congress report again proposed to “establish a fertility support policy system and implement a national strategy to actively cope with population aging [4]”.

With a strengthening aging trend and the relaxation of fertility policies, more three and even four-generation family structures have emerged at the micro-level [5]. Such a “sandwich-like” structure among ordinary families creates their own endowed resources, limited by family income. They must rebalance the allocation of internal resources given the increasing pressure to support older adults while raising children [6]. The process of redistributing resources can affect human capital investment in children, which can, in turn, lead to either intergenerational cooperation or conflict within the family [7,8].

Existing research has examined both household decision-making and labor time, although there is some controversy over labor time, arguing that older people living with children assist with childcare and household responsibilities and that intergenerational cooperation exists in families [8,9]. However, general studies have confirmed high intergenerational competition between household members in their human capital investment [7,10]. As aging begins to impact more family members, the increase in family resources for old age could lead to intergenerational competition as the main driver of intergenerational relationships in the family [11,12]. However, literature has rarely addressed the competition between the burden of old age and the pressures of childcare within such “sandwiched” families from an intergenerational conflict perspective. Few studies have analyzed how human capital investment decisions are coordinated across multiple subjects and among family members.

Therefore, we aimed to construct a virtual “sandwiched” family structure that includes a population of older adults and educated adolescents to simulate a family’s educational investment decisions. In doing so, we determined whether families’ future human capital investments are based on the conflict or cooperation between generations. The process design was as follows. First, we used panel fixed effects regression equations with household-, head-of-household-, and province-level control variables to initially determine the extent to which aging and childcare pressures affect a household’s educational expenditures [13,14]. Subsequently, we tested the robustness of the baseline regression results and considered the key effects of urban-rural factors, household aging, and household income on household education investment decisions [14,15,16]. Then, by introducing multi-agent simulations, dummy households and their respective key members were generated to analyze the differences in the changes in education investment for households with different membership structures under the risk of income changes and household resource constraints.

Our study contributes to the current literature in the following ways: First, we set control variables in multiple dimensions of household, head of household, and province, and added urban-rural factor, household aging difference factor, and income factor in a subsample regression to analyze the dual pressures of old age and childcare on household investment in education to make our findings robust and reliable. Second, we introduced a multi-agent simulation approach to reproduce the household’s educational investment decision-making process. This measure visually illustrates this process given constrained resource endowments, providing us with the possibility to observe the operations in real household decisions. Although some literature has argued that aging and childcare pressures have predominantly conflicting effects on households’ future human capital investments [17,18,19], the focus is primarily on the causal relationship between the three and there is a lack of discussion on a way to reproduce the decision-making process. Third, our study used a dynamic analysis approach to model changes in household income, providing a discussion on the magnitude of the effects of income and membership structure on household educational expenditures.

The remainder of this paper is organized as follows: Section 2 provides a brief overview of related literature. Section 3 describes the data set and variables, followed by our analytical methods. Section 4 presents the empirical results, Section 5 discusses the results of our simulation, and Section 6 concludes the paper.

## 2. Literature Review

Studies related to micro-family educational investment decisions have focused on the effects of childcare stress, the burden of aging, and the effects of intergenerational relationships between the two.

The family childcare burden’s impact on family investments in educational human capital can be traced to Becker’s (1960) theory of child quantity and quality [20]. This theory suggests that family size is a key cost factor when realizing a child’s potential and that this cost increases when the number of children remains constant. In empirical studies, value is placed on factors such as educational attainment or health status, and most studies demonstrate that family size has a significant, negative effect [21,22] especially regarding the number of children [23]. The more the children in a family, the lower the family resources allocated to each child, and the less advantages they are afforded [24]. Additionally, important factors influencing the investment of educational resources within the family include the family’s socioeconomic status [25,26], the child’s innate endowment [27], the level of parental involvement [25,28], and the child’s gender [29,30]. Overall, families must expend significant resources and input into their children’s education. The pressures involved in raising a large family can overwhelm family resources for education.

Most studies on aging’s impact on human capital educational investments have been conducted at the macro-level. They have focused on the primary effects that population policies exert on economic growth––on labor productivity in general and on human capital in particular [31,32,33]. Few studies systematically and empirically analyze the mechanisms and effects of population aging on households as an important subject of investing in human capital. In developed Western countries, established and more comprehensive social security and educational systems have replaced micro-family decision-making. They provide old age services and educational support [34], coupled with a lack of cultural emphasis on family obligation to provide support [35]. Hence, foreign literature rarely analyzes the micro-family-level effects [35,36]. Families in modern society often age before attaining prosperity, and they must fill the supply and demand gap between social security mechanisms and old age needs. As families balance investments in old age and education, aging often overtakes the family’s investments in educational resources, generating intergenerational negotiations, competitions, compromises, and cooperation within the family for human capital investments [9,37].

**Figure 1:**
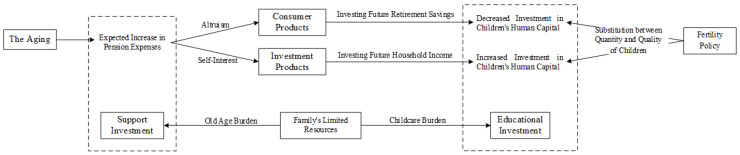
The decision-making mechanism of households’ human capital investments.

The impact of the dual pressures of old age and childcare on human capital educational investments revolves around the motivation of intergenerational relationships [38]. The existence of both altruistic and self-interested motives between generations leads to a dynamic balance between these investments and returns [39,40]. This results in the intergenerational nature of family human capital investments: parents invest in their children’s education expecting that they in return will provide them healthcare and social security human capital expenditures in their old age [41,42]. Figure 1 illustrates the decision-making mechanism of households’ human capital investments. Out of altruism, decision-making members view their children’s human capital as a durable good [43]. As society ages and the pressure to support the future population of older adults increases, households must exchange resources among family members of different ages when the household income is constrained [19]. When anticipated old-age expenditures increase, households may favor the use of savings to meet future old-age expenses, which also implies that these expenses will overtake any investments in children’s human capital [44].

Driven by self-interest, decision-making members may also perceive their children’s human capital as an investment [45]. When the expected pressures of old age increase, households may increase investments in their children’s education to generate greater future returns and protect their needs later in life [44]. These children still need their parents to provide more human capital investments in their education and career development owing to increased social competition [46,47]. From the older adults’ perspective—both subjectively given the family decision-making role, and objectively given the time needed for such labor—the increased need for care among older adults compels families to expect to obtain more future income [12,48]. Therefore, we have formulated the following research hypothesis:

**H1.**  *The dual stresses of family retirement and childcare both significantly and negatively relate to the family’s educational expenditures.*

Considering that a household’s income significantly impacts its consumption, several studies have found that household income significantly and positively affects a household’s educational expenditure when such expenditures are limited [48,49,50]. In addition, urban-rural factors and the degree of old age and aging likewise affect household education expenditures [14,34,51]. Hence, we also propose the following hypothesis:

**H2.**  *Families in urban and rural areas, at different levels of aging, and with different incomes, are significantly and differently affected by the dual pressures of old age and childcare.*

**H3.**  *Increased income can mitigate how these dual stressors overtake household educational expenditures.*

## 3. Data Variables and Methods

### 3.1. Data

The project data used in this study are from the China Family Panel Studies (CFPS) conducted by the China social science survey center (ISS) at Peking University. The project began in 2010 with an ongoing multi-stage, stratified sample of households in the 25 provinces, autonomous regions, and municipalities directly under China’s central government. The survey covered all members of the sampled households, including village status, household status, and individual adult and child status. This study used three years of data from 2014, 2016, and 2018, with a sample of 42,183 households. Members of the household sample were required to include older adults, adolescents younger than age 19, and intermediate-generation parents. The family-level variables were obtained from the family questionnaire pool merged with the family relationship pool using the family identification as the linkage variable. Individual-level variables for the household head were obtained from the household questionnaire pool merged with the individual self-response pool using the household economic decision-maker identification as the linking variable. Province-level variables were obtained from the China City Statistical Yearbook and the provincial statistical yearbooks. After excluding some sample observations with missing variables and the outliers of key variables, the final household sample comprised 5677 households.

### 3.2. Variables and Descriptions

#### 3.2.1. Dependent Variable

This study’s dependent variable is the household’s investment in educational human capital, which was measured by total household educational expenditures after referring to existing studies [17]. The main components include nine expenses: school fees, school meals, accommodations, books, school choice fees, school bus fees and transportation costs owing to study, educational software, extracurricular tutoring fees, and other expenses. In addition, the study also selected per capita educational expenditures and the household’s non-compulsory educational expenditures to replace the dependent variable to test the robustness of the regression results [14].

#### 3.2.2. Independent Variables

The core independent variables are the burden of old age and the stress of caring for young children. This study considered existing research [19] to measure the burden of old age as the number of persons aged 60 or older in the household as a proportion of the total number of persons in the household; the stress of caring for children was measured by the number of young people under age 19 in the household as a proportion of the total number of persons in the household. The number of people over 60 years old and the number of young people under 19 years old in the household were selected simultaneously to test the robustness of the regression results [12].

#### 3.2.3. Control Variables

Existing studies demonstrate that households’ educational investments are affected by three factors: the household level, the head-of-household level, and the macro level of the province [13,14,19]. The household-level control variables include the number of household members, net household income, and total household assets. Since the CFPS data do not reveal the household head’s identity, this study relied on the “financial responder” question in the household survey questionnaire to identify information about the household head, which primarily includes their age, gender, and years of education. The provincial macro-level control variables include the education expenditure component of general public budget expenditures, disposable income per capita of all residents, and the number of community service institutions and facilities for older adults. Among the information provided in this question, urban status and gender are the dichotomous variables.

#### 3.2.4. Descriptive Statistics

Table 1 presents detailed explanations of all the previously noted variables. The average annual total education expenditures of the households sampled is ¥5744.55 and the average annual non-compulsory education expenditures is ¥1044.04. The standard deviation of these indicators is greater than the mean, indicating that different households have significantly varying characteristics in their educational expenditures. In terms of the burden of care of older adults, the average number of people over 60 years old in a “sandwiched” household is approximately 29.8% of the total number of people in the household. Regarding the pressure of raising young children, the average number of young people (under age 19) in a household is approximately 31% of the total number of people in the household. At the household level, approximately 42.9% of the households in the sample are urban, with an average household size of five members; nearly 21.8% of those have pension insurance. At the head-of-household level, the head of the household is 50 years old on average, and men (55.2%) tend to make the household’s economic decisions.

### 3.3. Methods

#### 3.3.1. Regression Analysis

The first question considered was whether the household burdens of old age and pressure to raise young children usurp the household’s educational investments. We constructed the following benchmark in Equation (1) to control for the household-, head-of-household-, and province-level characteristic variables and estimate them using a panel fixed-effects regression model:

(1) $$edu_{it}=\alpha_{0}+\alpha_{1}ratio\_60_{it}+\alpha_{2}ratio\_19_{it}+\beta X_{it}+u_{i}+v_{t}+\varepsilon_{it}$$

where $edu_{it}$ represents the total educational expenditures per household per period; $ratio\_60_{it}$ represents the proportion of the number of older adults over age 60 in each period to the total people in the household; $ratio\_19_{it}$ represents the proportion of the number of adolescents under age 19 in each period to the total people in the household; $X_{it}$ denotes a series of control variables, such as the household level and head-of-household level; $u_{i}$ and $v_{t}$ represent household individual fixed effects and time fixed effects; and $\varepsilon_{it}$ is the error term containing the individual and temporal dimensions. Table 1 provides a detailed explanation of each variable. This study analyzed the data using Stata software (Version 17).

#### 3.3.2. Multi-Agent Simulation Model

After clarifying the impact of the dual pressures of old age and childcare on household educational expenditures, we used a multi-agent simulation to reduce the decision-making process for these expenditures. We aimed to observe the differences in decision-making among households with different members and resource endowments.

**Figure 2:**
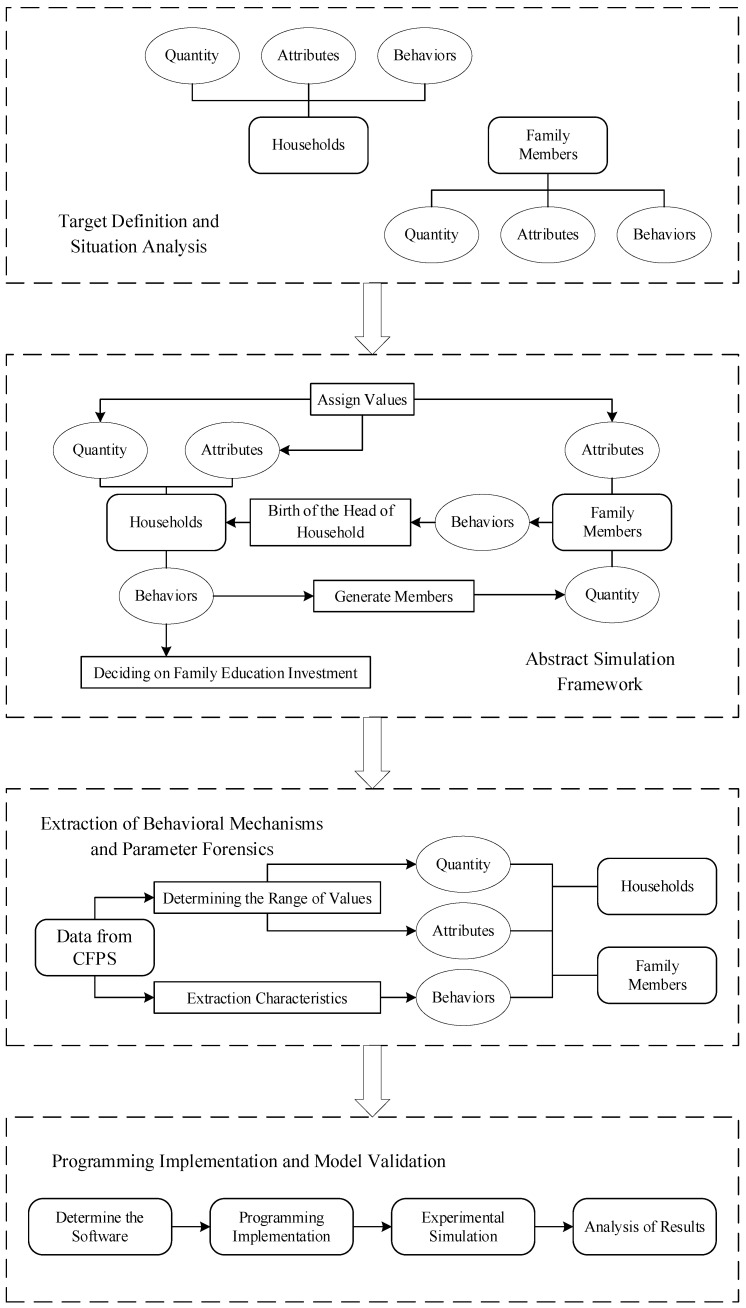
The multi-agent simulation process.

The multi-agent simulation, or multi-agent system (MAS) simulation, solves large-scale complex problems beyond the capability of a single subject by forming an interactive group of multiple subjects. The MAS is a complex system, in which each individual is independent of the other and has its own set of strategies. Within the system, each individual evaluates their own state according to environmental changes and feedback mechanisms and decides to either change the state of their attributes or to react. The interactive relationship between individuals provides an evolutionary process for complex systems. Through overall emergence, this ultimately achieves the main goal of the system or affects the external environment of the system. A computer simulation can observe this duality of internal and external strategies and offer a reference for the information value in real system operations. In this study, we constructed three-generation “sandwich” families with different resource endowments and membership structures through a multi-agent simulation approach. These simulated households form their own educational investment decisions based on their resource endowments. Simultaneously, we dynamically analyzed the changes in educational investment decisions of households with different membership structures with changes in income. Figure 2 illustrates our process for the multi-agent simulation.

## 4. Empirical Results

### 4.1. The Impacts of Old Age Burden and Childcare Stress on Household Educational Expenditures

Before regression analysis, this study first determined the chosen model by Hausman test, which showed that the original hypothesis was rejected at the 1% significant level; thus, the fixed-effects model was used as the main model. The empirical model was constructed according to Equation (1), combining the three-period panel data of CFPS and controlling for both year fixed effects and household fixed effects to test the effects of old-age burden and childcare stress on household education expenditures. To investigate the effects of each control variable on household education expenditures in greater detail, this study gradually added different levels of control variables in the estimation process. Table 2 reports the results of the benchmark regressions of the burden of old age and childcare on the family’s human capital educational investments obtained using a double fixed effects model.

Regression Model (1-1) regresses the shares of older adults and adolescents as the primary explanatory variables, and both regression coefficients are found to be negative. The regression coefficients for the number of adolescents are significant at the 1% level, indicating that the family’s pressure in raising young children negatively influences household educational investments to some extent. Regression Models (1-2) and (1-3) demonstrate that the correlation between household educational investments and the household’s proportion of people aged 60 or older and the proportion of youths aged 19 or younger does not fundamentally change (after gradually controlling for household-, head-of-household- and province-level control variables). Both are significant at the 5% level. Controlling for household fixed effects, year fixed effects, and all-level control variables reveals that a 1% increase in the number of people over 60 in the household would, on average, cause the household to reduce its investment in children’s education by ¥44.04. Similarly, a 1% increase in the number of youths under 19 would, on average, cause the household to reduce its investment in children’s education by ¥28.78.

In summary, the base regression results further validate the previous theoretical derivation. With an increasing trend of aging and increase in family size, the older adult burdens and childcare pressures on families do overshadow their educational human capital investments to a certain extent. Additionally, the negative effect from the older adult burdens is more intense.

### 4.2. Robustness Tests

To ensure the robustness of the previous empirical estimation results, this study conducted robustness tests by replacing variables and shortening the sample interval. Regarding the dependent variables, the total education expenditure of the household only measures part of the subjective choice of the household owing to the policy support of nine-year compulsory education in China, which obliges parents to send their school-age children to compulsory education, and the government covers children’s school fees at this stage. In our study, we replaced the total educational expenses with “non-compulsory educational expenses” to conduct a robustness test to more scientifically characterize the effect of old-age burden and childcare stress on household educational expenses, with the results shown in Table 3 Regression Model (2-1). We also replaced the explanatory variable with “educational expenses per capita” for robustness testing, and the results are reported in Table 3 Regression Model (2-2). The independent variables were replaced with the number of people over 60 years old and the number of young people under 19 years old in the household for robustness tests, and the results of the re-regression are presented in Model (2-3). Furthermore, this study shortened the sample interval for robustness test and selected the data of the 2016 household sample to reestimate the regression of dual stress on household educational expenses, with the results reported in Model (2-4).

By comparing Regression Models (2-1) and (2-2) of Table 3 with Table 2 of the baseline regression results, we find that the significance of the effect of the older adult burdens and childcare pressures on household non-compulsory educational expenditures and per capita educational expenditures decreased after replacing the dependent variables. However, the direction of the effect did not change, and household educational expenditures decreased significantly as the dual pressures increased. The robustness results of replacing the independent variables show that the number of people over 60 years old and the number of young people under 19 years old in the household also negatively impact household education expenditure, but the significance and regression coefficients have decreased. Shortening the sample interval for further analysis, the results in Regression Model (2-4) show that the significance level of the independent variables did not decrease, and the positivity and negativity of the regression coefficients did not fundamentally change. Therefore, we can confirm that the results obtained in the baseline regression are robust and that dual stress has a negative impact on household education expenditures.

### 4.3. Heterogeneity Analysis

To obtain more comprehensive and detailed estimates, this study further examined the effects of dual stress on education expenditures across urban and rural areas, different levels of aging, and households in different income brackets.

To explore the urban-rural differences in households’ investment in educational human capital, group regressions are conducted with rural and urban areas, and the results are reported in Table 4 Regression Models (3-1) and (3-2). The regression results show that aging negatively impacts investment in education for both rural and urban households, and the estimated coefficients are more significant and larger in absolute value for rural households. Regarding childcare pressures, an increase in the proportion of youth aged 19 or younger negatively affects educational investment in rural households but promotes it in urban households.

Considering the impact of different levels of household aging on household investment in education, we divided the households into two groups of samples—lower and higher age—based on the age distribution of older adults in the data, using 65 years as the grouping criterion for grouping regressions, with the results reported in Regression Models (3-3) and (3-4). Significant differences were found in the impact on households by age. The regression estimates show a negative crowding out of household investment in education by aging in both the lower and higher age groups, and that the negative shock is more severe for households in the higher age group. In terms of easing childcare policies, the increase in the proportion of youth aged 19 or younger manifests itself as a crowding out of family investment in education in households in the higher age group, but as a positive boost in households in the lower age group.

Among all household control variables, household income is susceptible to external influences and is volatile in the short term. This study investigates the extent to which families with different incomes are affected by their investment in educational human capital. It classifies families as “low-income”, “middle-income”, and “high-income” based on their quantile of net family income. The regression results corresponding to each other are detailed in Table 4 Regression Models (3-5), (3-6) and (3-7). The burdens of old age and child-rearing have slightly different effects on educational investments among households in different income brackets. The regression estimation results reveal that aging negatively affects educational investments, but the estimated coefficients are more significant and have a larger absolute value in low- and middle-income households. Hence, the investments in educational human capital among low- and middle-income households are susceptible to the adverse effects of the increasing burden of older adults. In terms of childcare-easing policies, the substitution effect between the number and quality of children is more pronounced in low- and middle-income households, while an increase in household size in high-income households positively contributes to children’s quality. Among the low- and middle-income households with significant effects, middle-income households are more negatively affected by the burdens of older adult care and childcare.

### 4.4. The Simulation Model and Implementation of Family Educational Investment Decisions

This study considers these basic regressions and uses NetLogo 6.1.1––a basic software program suitable for a multi-agent simulation analysis––to simulate the process of micro-household investments in educational human capital under the dual pressures of aging and childcare. In this study, a three-generation “sandwiched” household is constructed based on the assumption that each household consists of adults over age 60, adolescents under age 19, and intermediate parents. We then simulate the household’s decision to invest in education with different resource endowments. Table 5 presents the simulation model’s specific parameters (province-level variables were not included, considering the consistency of the variables controlling for simulated households).

**Figure 3:**
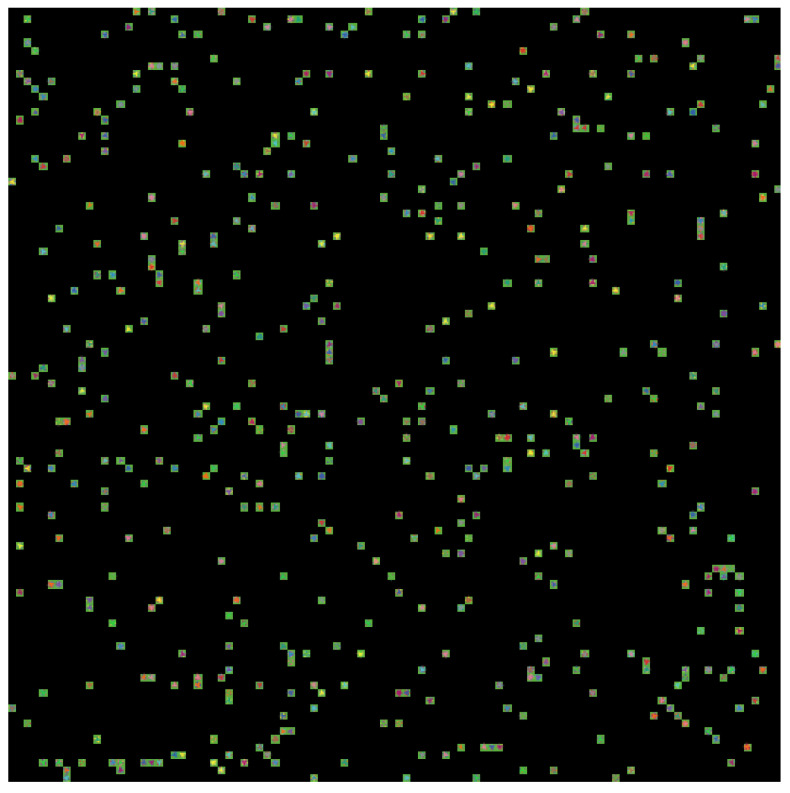
Simulated working area.

**Figure 4:**
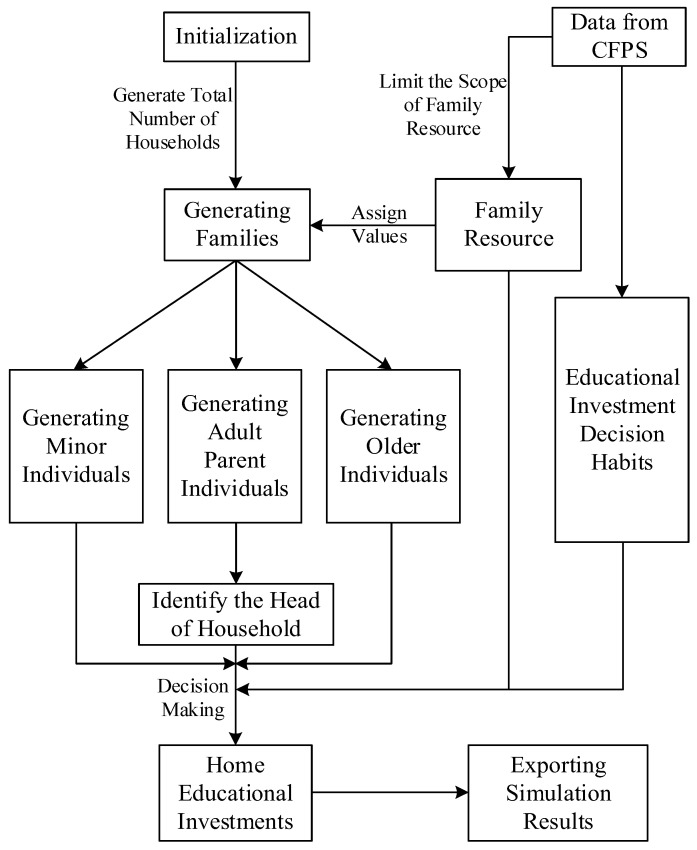
Family decision-making process from the multi-agent simulation.

In the model’s initialization process, the household resource endowment is set based on the CFPS database, and the corresponding variables’ range of values are noted by the range of value changes. The obtained resource endowment determines how the households in this simulation generate family members and indicates the head of household candidates; furthermore, household educational investment decisions are made based on the education investment habits formed by the regression results in Table 2 (Model 1-4). Figure 3 presents the simulation working area, whereas Figure 4 illustrates the main process. In Figure 3, the green squares represent each family’s location, as measured by horizontal and vertical coordinates; the small markers with colors represent the members generated in the family. Table 6 displays some of the households’ resource endowments and simulated investment results.

### 4.5. Simulation Implementation of Household Educational Investment Decisions under Income Differences

**Figure 5:**
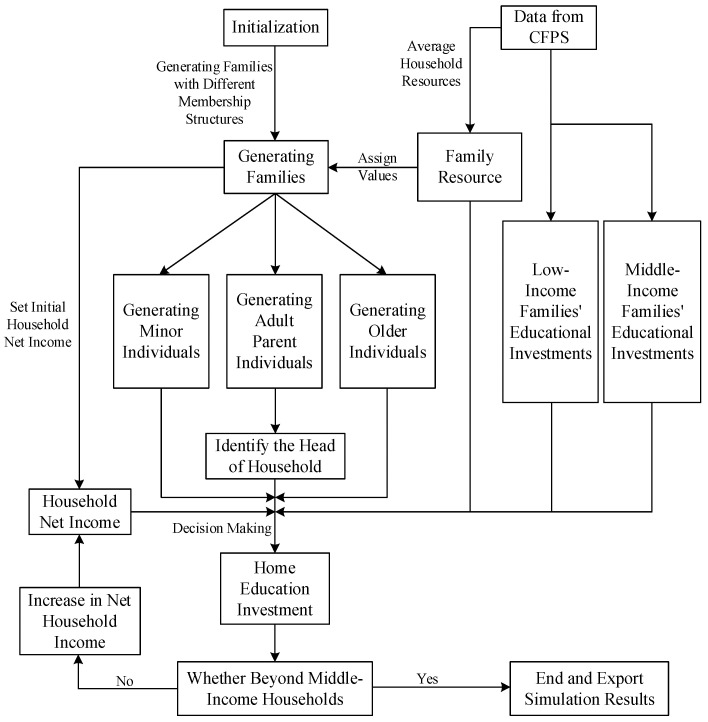
Simulation process of education investment for low- and middle-income households under income growth.

The income disparity analysis demonstrates that education in low- and middle-income households is vulnerable to the negative effects from the burdens of old age and childcare. These effects are both significant and heterogeneous, and income changes are the most vulnerable factor in the short term in household resource endowments. This study attempts to explore the differences in educational investments owing to the different membership structures in low- and middle-income households. This is based on a model of household educational investment decisions. Table 7 shows that the simulated households are endowed with average household resources to not only generate common household structural types as noted in the CPFS database, but also to make investments based on low- and middle-income households’ educational investment habits. Specifically, these households’ initial net household income begins at the low-income level and increases by ¥5000 net income per cycle until they exceed the middle-income bracket. Figure 5 illustrates this process.

**Figure 6:**
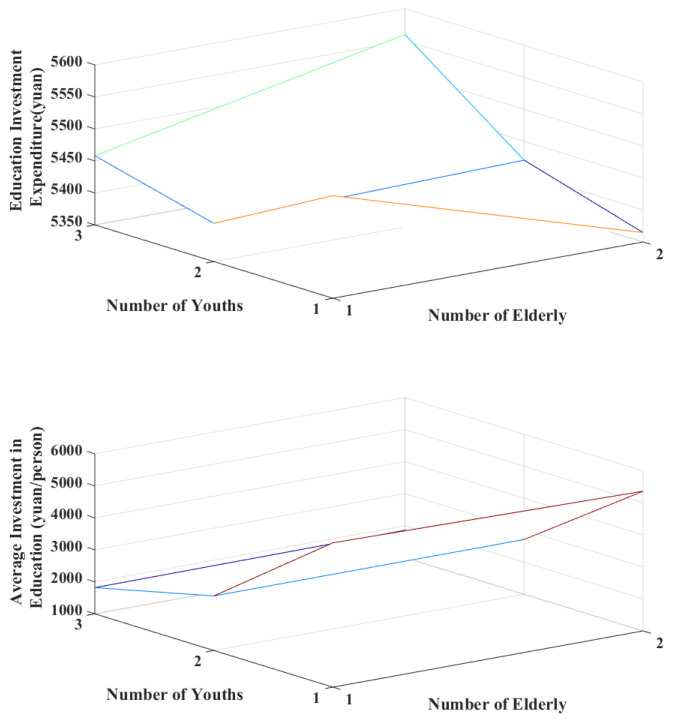
Simulation results of educational investments for low-income households.

**Figure 7:**
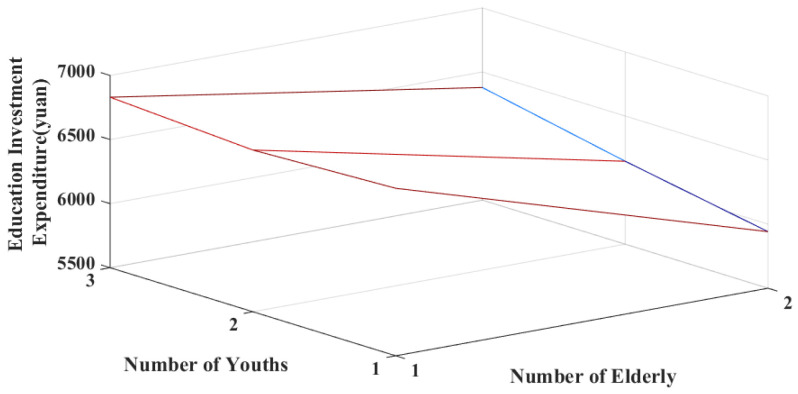

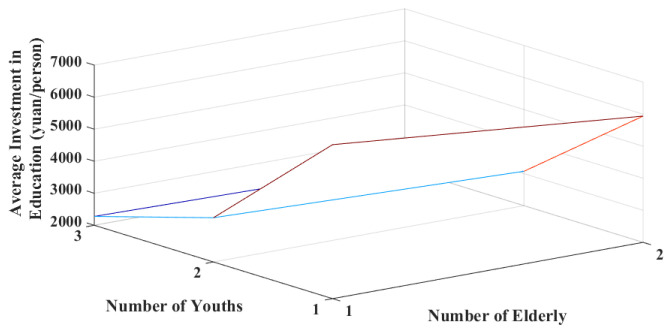
Simulation results of educational investments for middle-income households.

Figure 6 and Figure 7 consider the previously noted settings to partially reveal the simulation results of households’ educational investments. Figure 6 presents the variations in educational investments among low-income families, with members composed if the family’s annual net income is ¥25,000. Figure 7 illustrates the variation in educational investments among middle-income families and the members’ age structure if the family’s annual net income is ¥55,000. Low-income households have less variation than middle-income households in both educational investment expenditures and per capita educational investment expenditures; the means of both are also relatively lower. This is consistent with the previously described regressions for the different income classes. Among the personnel structure of households in the low-income bracket, educational investments are most affected by households with one adolescent and two older individuals, and two adolescents and one older individual. From the person-ratio perspective, families with a substantial older adult burden and less childcare pressure and those with high childcare pressure and less of an older adult burden should focus more on the family’s future human capital investments. Among the personnel structures of middle-income households, education expenditures are most profoundly affected by households with one youth and two older individuals. From the household composition perspective, households with a more substantial older adult burden and low childcare pressure need to focus more on their future human capital investments. Comparing the per capita education investments of low- and middle-income families indicates that the most affected families are those with high childcare pressures. These families must focus more on the unequal distribution of family education resources among children.

**Figure 8:**
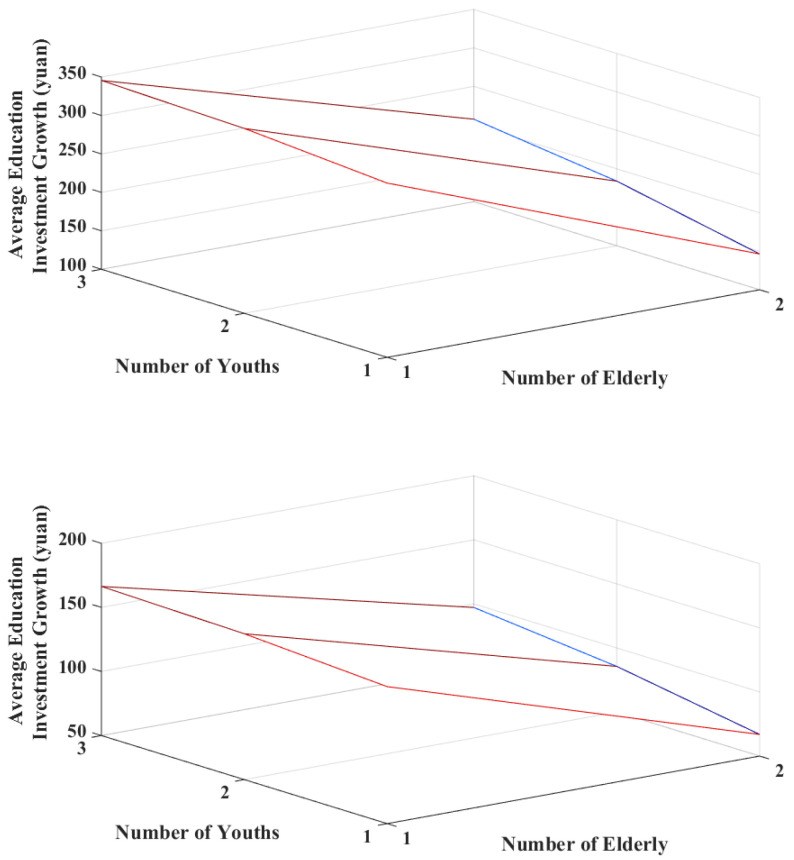
Growth in average educational investments, with increasing income for low- and middle-income households.

Regarding income growth, Figure 8 presents the growth in average educational investments for households of different income compositions for different persons with each ¥5000 increase in their annual net income. Comparing the left and right graphs demonstrates that the curved surfaces for middle-income households change less and have relatively lower means. Hence, households relieving a certain amount of life stress reach a certain saturation in their educational investments and are not susceptible to income changes. Additionally, the increase in income further increases the educational investment for both low- and middle-income households, with the effect being most pronounced in households with high childcare and low older adult burdens and slowest among households with a low childcare and high older adult burden.

## 5. Discussion

This study aimed to introduce a new approach to model the impacts of households’ older adult and childcare burdens on household educational expenditures. Our study contributes to the current literature by constructing virtual families through a multi-agent simulation approach, while discussing the changing impact of these dual stressors on households’ educational expenditures from two dynamic perspectives: changes in household income and the household staffing structure. Such a measurement approach has two advantages. On the one hand, it can reflect families’ actual decision-making process in weighing their educational investments under the dual pressures of old age and childcare. On the other hand, families with different membership structures intuitively exhibit differences in the degrees of impact that can help specific families protect their future human capital. Our methodology is applied to micro-household human capital investment decisions under China’s antiquated and relaxed fertility policies. Our descriptive statistical analysis reveals that a proportion of Chinese micro-households are indeed affected by high older adult or childcare burdens and that considerable variations exist in these households’ educational expenditures. Subsequently, we estimated these burdens’ impacts on household educational expenditures. We observed that both burdens significantly and negatively affect household educational expenditures, although the negative crowding-out effect from household aging is more intense. Moreover, the above findings remain robust after robustness tests, such as replacement variables and shortened sample intervals, were performed. Thus, Hypothesis 1 is supported.

As Li noted, exploring the burdens of aging and child support, both of which have a crowding-out effect on households’ human capital investments, is necessary [17]. However, there are some differences regarding the impact of these dual pressures on urban and rural areas, on different levels of aging, and on different income households, thereby supporting Hypothesis 2. For both urban and rural areas, the negative impact of the burden of older adults is observed in all households and the estimated coefficients are highly significant; however, the increase in the burden of child care in households shows a depressive effect on household investment in education in rural areas, but a facilitative effect in urban areas. The main reason for this is that urban areas have more educational resources and a rich economic and cultural life [14]. In this atmosphere, urban families pay more attention to the education and training of their family children, which to some extent protects the family education expenditure from the pressure of childcare.

With respect to the degree of household aging, the older adult burden exhibits a negative crowding-out effect for both households with lower-aged older adults and those with higher-aged older adults, but its coefficient is smaller in absolute value for the lower age group. By contrast, the increase in the burden of childcare in households shows a boosting effect on household investment in education in the lower age group, but a dampening effect in the higher age group. The fact that the lower age group is still capable of working and self-care and helps with childcare and household chores [8] is reflected in the regressions as the education expenditure is less affected by the burden of older adults in the household group with the lower age group. Furthermore, because of the intergenerational partnership described above in the lower-aged older adults household group [9], for families, some of the children’s expenses other than education are replaced by the labor time of older adults [12], which in turn protects the household’s educational expenses.

The household income component also significantly affects education expenditures, leading to different educational expenditure-related decisions for households with varying incomes [48,51]. First, the negative impact of a substantial older adult burden is observed across income groups, and the estimated coefficients are highly significant among low and middle-income households. Second, the substitution effect between the number of children in the family and opportunities afforded to them is more pronounced in low- and middle-income households, while an increase in family size in high-income households positively contributes to these opportunities. Performance also significantly varies among households with different incomes, with some studies observing that higher-income households have more disposable income and a richer endowment of household resources that allows them to face dual pressures on resources more comfortably [19,25]. Moreover, decision-makers in higher-income households tend to invest in human capital out of self-interest [39,43], view educational expenditures as an investment in their children, and increase expenditures to maintain or even increase household income in the future.

We used a multi-agent simulation approach to further investigate the changes in educational investment decisions of households with different membership structures when faced with the risk of income changes. First, households in the middle-income bracket are more vulnerable to the burdens of old age and the pressures of raising young children, consistent with the subsample regression results. The thresholds for older adult and childcare criteria are relatively low among low-income households [26]. Educational investment expenditures are relatively necessary for the household but less subject to fluctuations in the composition of household members. Second, both low and middle-income households’ per capita educational investments are most affected by high childcare pressures. This may lead to an unequal distribution of educational resources among children [23,30]. Finally, Hypothesis 3 is supported by the fact that household income growth has a catalytic effect on households’ educational investments, consistent with findings from existing literature [48,51]. However, the effects of such income changes on the growth of household educational investments are more pronounced in the low-income class. Furthermore, the crowding-out effect from a substantial older adult burden on the growth of household investments in future human capital owing to income growth is more significant than that of high childcare pressure.

Some limitations of this study should be noted here. First, we used dual independent variables to portray the burden of old age and the stress of caring for young children in the household, a setting that leaves us without effective instrumental variables for the time being, and the regression results have some endogeneity problems. Second, our simulation program for the family education investment decision process is in the preliminary stage of research, the experimental program is not perfect, and we only ran simulations for the effect of changes in family income. In the future, we will further improve the program and conduct further research on more factors.

## 6. Conclusions

This study incorporated panel data from the CFPS for 2014, 2016, and 2018 to investigate the impact mechanism and decision-making process of micro-level human capital investments. Our three-generation, direct line, “sandwiched” family functioned under the dual perspectives of older adult and childcare burdens. We also simulated the decision-making process involving households’ educational investments through a multi-agent simulation. This allowed us to examine the actual effects of dynamic income changes on the educational investments of households with common membership structures.

We discovered that China’s aging population and its relaxed fertility policy have a crowding-out effect on the human capital educational investments of “sandwiched” households, and that this effect differs markedly across urban and rural areas, different levels of aging, and different incomes. Our computer simulations revealed that families with high childcare pressures allocate more investments in human capital for family education than those with a high retirement burden. This is owing to their differences in resource endowments due to household income. However, high childcare pressures may also lead to an unequal distribution of educational resources among children. Moreover, the increase in household income mitigates the crowding-out of educational resources to some extent due to the double pressure. However, the boosting effect of income growth initially diminishes with further income growth. In this process, the weakening trend is also further exacerbated by the comparatively higher burden of old-age care.

Our findings have certain policy implications and practical value. In the context of aging and relaxed fertility policies, we should continue to adhere to the policy of “strengthening the country through education,” pay deep attention to the quality of education in less developed regions, allocate public education resources, and improve the relevant education guarantee system. This is especially valid for disadvantaged rural and low and middle-income families to guarantee their children’s right to receive a compulsory education. Simultaneously, focusing on the membership structure within the family is also crucial. A new public policy regulatory system for intergenerational interactions among families with a specific income and membership structure could be built. This will help families rationalize the use of borrowing and saving to optimize consumption, reduce the pressure of uncertainty on family investment in education, enhance their investment in education, and protect their quality of life. In addition, we should also increase the promotion of residents’ pension insurance and medical insurance, improve the quality of insurance, and encourage commercial insurance to develop various types of insurance for older adults to enhance the living and medical protection capacity of older adults in the family and help actively resolve the intergenerational conflict between family retirement expenses and childcare investment.

## Figures and Tables

**Table 1:** Variables’ statistics.

Variable	Description	Mean	SD	Min.	Max.
Dependent variable					
Education	Total family educational expenses (in Chinese yuan)	5744.549	7548.048	0	80,000
P_education	Educational expenses per capita (in Chinese yuan)	1079.660	1433.175	0	17,500
Non-compulsory education	Family educational expenses except for compulsory education (in Chinese yuan)	1044.042	4022.227	0	104,000
Independent variables					
Ratio_60	Number of people over age 60 in the household as a proportion of the total number of people in the household	0.298	0.090	0.083	0.500
Ratio_19	Number of adolescents under age 19 in the household as a proportion of the total number of persons in the household	0.310	0.095	0.200	0.750
Num_60	Number of people over age 60 in the household	1.605	0.489	1	2
Num_19	Number of adolescents under age 19 in the household	1.730	0.732	1	6
Household-level control variables					
Urban	Urban = 1, rural = 0	0.429	0.495	0	1
Familysize	Total number of families	4.940	1.057	2	10
Ratio_insurance	Number of people in the household with pension insurance as a proportion of the total number of people in the household	0.218	0.161	0	0.800
Income	Annual net household income (in Chinese yuan)	68,735.747	41,786.592	14,360	224,000
Asset	Household net worth (in Chinese yuan)	442,980.393	412,573.785	59,950	2216,250
Head of household-level control variables					
Age	Age of the head of household	50.311	13.567	25	81
Age_aq	Square of the age of the head of household	2715.246	1405.580	625	6561
Gender	Male = 1, female = 0	0.552	0.497	0	1
Eduy	Head of household’s years of education	7.211	4.237	0	19
Provincial macro-level control variables					
Public education	The education expenditure component of local general public budget expenditures, taking the logarithm (in Chinese million yuan)	15.859	0.559	14.172	17.050
PCDI	Per capita disposable income of all residents by province (in Chinese yuan)	21,843.407	7850.256	12,184.700	64,182.602
Service	Number of community service institutions and facilities for older adults by province	1201.616	736.876	212	3409

**Table 2:** The pressure of raising a child and its impact on household educational investments.

	(1-1)	(1-2)	(1-3)	(1-4)
	Education	Education	Education	Education
Independent variables				
Ratio_60	−6454.839 *** (931.930)	−4631.999 *** (1168.306)	−4719.331 *** (1163.702)	−4403.619 *** (1160.648)
Ratio_19	−7093.002 *** (999.371)	−3305.775 *** (1079.626)	−2672.285 ** (1045.572)	−2877.578 *** (1051.063)
Household-level control variables				
Urban	—	298.021 ** (127.934)	202.056 (124.761)	207.035 * (125.489)
Familysize	—	568.653 *** (115.453)	567.868 *** (115.497)	551.937 *** (114.982)
Ratio_insurance	—	1996.202 *** (557.168)	2024.486 *** (557.089)	1500.791 *** (561.014)
Income	—	0.002 (0.001)	0.002 (0.001)	0.002 (0.001)
Asset	—	0.001 *** (0.000)	0.001 *** (0.000)	0.001 *** (0.000)
Head-of-household-level control variables				
Age	—	109.070 * (63.909)	104.469 (63.705)	105.596 * (63.436)
Age_aq	—	−0.683(0.639)	−0.672 (0.637)	−0.680 (0.634)
Gender	—	−1190.351 *** (248.047)	−1029.026 *** (251.978)	−1034.739 *** (250.929)
Eduy	—	267.277 *** (31.977)	249.711 *** (30.649)	251.462 *** (30.559)
Province-level control variables				
Public education	—	—	−493.661 (342.991)	−1173.016 *** (339.348)
PCDI	—	—	0.087 *** (0.029)	0.070 ** (0.030)
Service	—	—	0.338 * (0.175)	0.846 *** (0.189)
_cons	10,320.525 *** (515.635)	−483.629 (1814.690)	5231.304 (5036.530)	7587.611 (5029.501)
Family fixed effects	Controlled	Controlled	Controlled	Controlled
Year fixed effects	Uncontrolled	Uncontrolled	Uncontrolled	Controlled
Number of Obs.	5677	5677	5677	5677

Note: ***, **, and * indicate significance at the 1%, 5%, and 10% levels, respectively; robust standard errors are noted in parentheses.

**Table 3:** Robustness test results.

	(2-1)	(2-2)	(2-3)	(2-4)
	Non-Compulsory Education	P_Education	Education	Education
Independent variables				
Ratio_60	−4060.958 * (2251.026)	−1399.912 *** (230.323)	—	−7032.387 *** (2377.285)
Ratio_19	−3208.673 * (2148.663)	−402.525 * (215.364)	—	−4555.578 ** (2217.509)
Num_60	—	—	−1184.210 *** (298.014)	—
Num_19	—	—	−382.315 * (239.358)	—
Household-level control variables				
Urban	1237.493 *** (303.117)	46.003 (30.354)	197.548 (160.921)	138.102 (301.136)
Familysize	455.416 ** (222.950)	−63.502 *** (23.090)	1077.199 *** (169.752)	631.735 *** (225.757)
Ratio_insurance	4630.436 *** (1323.059)	284.356 ** (120.796)	2093.469 *** (639.491)	2678.572 (1635.577)
Income	0.002 * (0.001)	0.000 *** (0.000)	0.002 *** (0.001)	−0.000 (0.001)
Asset	0.001 *** (0.000)	0.000 *** (0.000)	0.001 *** (0.000)	0.001 *** (0.000)
Head-of-household-level control variables				
Age	213.684 * (126.050)	27.333 ** (12.737)	104.585 (67.488)	10.201 (118.755)
Age_aq	−1.441 (1.216)	−0.204 * (0.123)	−0.651 (0.653)	0.392 (1.156)
Gender	−1669.062 *** (443.830)	−225.619 *** (47.111)	−1040.677 *** (249.715)	−1543.565 *** (462.283)
Eduy	457.630 *** (55.744)	54.594 *** (5.793)	256.992 *** (30.733)	211.428 *** (57.124)
Province-level control variables				
Public education	−1760.508 *** (543.749)	−227.808 *** (59.430)	−523.749 * (314.907)	−1913.525 *** (614.652)
PCDI	0.172 *** (0.025)	0.022 *** (0.003)	0.090 *** (0.016)	0.129 *** (0.029)
Service	1.578 *** (0.369)	0.120 *** (0.039)	0.353 * (0.208)	1.053 ** (0.462)
_cons	1989.991 (8623.174)	3698.398 *** (922.275)	3140.145 (4897.710)	29,787.003 *** (9523.310)
Family fixed effects	Controlled	Controlled	Controlled	Uncontrolled
Year fixed effects	Controlled	Controlled	Controlled	Uncontrolled
Number of Obs.	5677	5677	5677	1860

Note: ***, **, and * indicate significance at the 1%, 5%, and 10% levels, respectively; robust standard errors are noted in parentheses.

**Table 4:** Heterogeneity analysis results.

	(3-1)	(3-2)	(3-3)	(3-4)	(3-5)	(3-6)	(3-7)
	Education	Education	Education	Education	Education	Education	Education
Independent variables							
Ratio_60	−7277.258 *** (1678.098)	−5651.452 ** (2295.341)	−4719.728 *** (1581.741)	−6120.201 *** (1965.339)	−4653.414 *** (1445.953)	−5364.245 *** (1123.091)	−4623.383 * (2799.458)
Ratio_19	−3189.253 ** (1273.060)	1471.153 (2290.998)	2607.450 * (1512.793)	−4837.957 *** (1612.468)	−2158.799 * (1249.236)	−2159.367 ** (992.380)	2167.635 (3072.802)
Household-level control variables							
Urban	—	—	176.759 (204.826)	277.680 (242.103)	−86.188 (226.642)	−114.621 (159.440)	599.870 * (339.827)
Familysize	261.579 *** (95.246)	736.392 *** (217.125)	307.940 * (161.294)	805.274 *** (169.285)	339.675 ** (146.830)	382.408 *** (110.745)	557.204 *** (175.740)
Ratio_insurance	1487.787 ** (736.804)	1675.200 (1304.740)	984.031 (935.138)	1447.649 (1165.606)	2342.202 ** (970.234)	1360.221 ** (620.188)	2211.310 (1714.388)
Income	−0.000 (0.001)	0.003 *** (0.001)	0.006 *** (0.001)	0.001 (0.001)	0.027 * (0.014)	0.016 *** (0.006)	0.000 (0.001)
Asset	0.000 *** (0.000)	0.001 *** (0.000)	0.001 *** (0.000)	0.001 *** (0.000)	0.000 (0.000)	0.000 (0.000)	0.001 *** (0.000)
Head-of-household-level control variables							
Age	130.521 * (75.083)	36.901 (126.888)	128.740 (111.787)	245.486 ** (96.458)	193.523 ** (81.691)	210.071 *** (59.973)	−194.096 (171.715)
Age_aq	−1.039 (0.728)	−0.013 (1.221)	−1.140 (1.145)	−1.872 ** (0.900)	−1.739 ** (0.782)	−1.823 *** (0.581)	2.330 (1.656)
Gender	−488.846 (299.560)	−1495.704 *** (428.327)	−1100.436 *** (322.691)	−1094.097 *** (370.841)	−837.693 *** (320.684)	−650.066 *** (234.156)	−1545.392 *** (572.001)
Eduy	188.249 *** (36.993)	332.552 *** (54.776)	177.948 *** (39.632)	337.101 *** (45.859)	118.036 *** (39.508)	128.513 *** (29.505)	360.374 *** (74.914)
Province-level control variables							
Public education	−734.997 * (401.636)	−714.953 (527.223)	−304.471 (408.042)	−866.868 * (467.067)	−336.638 (462.317)	−517.632 * (311.519)	−617.549 (735.962)
PCDI	0.148 *** (0.026)	0.066 *** (0.023)	0.066 *** (0.022)	0.099 *** (0.023)	0.136 *** (0.038)	0.077 *** (0.022)	0.024 (0.029)
Service	0.205 (0.239)	0.704 * (0.379)	0.408 (0.262)	0.427 (0.318)	0.110 (0.244)	0.271 (0.184)	0.934 * (0.557)
_cons	10,044.435 * (6096.454)	8136.241 (8431.386)	1847.861 (6528.137)	7186.261 (7293.011)	1305.110 (6898.770)	4738.570 (4732.635)	12,514.016 (11,700.305)
Family fixed effects	Controlled	Controlled	Controlled	Controlled	Controlled	Controlled	Controlled
Year fixed effects	Controlled	Controlled	Controlled	Controlled	Controlled	Controlled	Controlled
Number of Obs.	3241	2436	2408	3269	1892	1892	1893

Note: ***, **, and * indicate significance at the 1%, 5%, and 10% levels, respectively; robust standard errors are noted in parentheses.

**Table 5:** Simulation of family property variables.

Variable	Description	Range of Values
Household		
Num. of family	Number of simulated households	(0, 500]
Urban	Urban and rural classification	Urban = 1, Rural = 0
Income	Household income (in Chinese yuan)	[14,360, 224,000]
Asset	Household net worth (in Chinese yuan)	[59,950, 2,216,250]
Householders	Head of household’s gender	Male = 1, Female = 0
Edu.	Household educational expenditures (in Chinese yuan)	The simulation results yield
Older adults		
Num. of older	Number of older adults generated by the household	1 and 2
Age	Age	[60, 100]
Gender	Gender	Male = 1, Female = 0
Eduy	Years of education	[0, 16]
Insurance	Whether participating in pension insurance	Yes = 1, No = 0
Intermediate-generation parents		
Num. of adults	Number of adult parents in the family	2
Age	Age	[20, 60]
Gender	Gender	Male = 1, Female = 0
Eduy	Years of education	[0, 19]
Insurance	Whether participating in pension insurance	Yes = 1, No = 0
Youth		
Num. of youth	Number of youths in the family	[0, 10]

**Table 6:** Selected households’ resource endowments and simulated investment results.

Fid	#/Older Adults	#/Youth	Urban	Income	Asset	Head of Household’s Age	Head of Household’s Gender	Head of Household’s Years of Education	Education
(51,46)	2	2	0	218,971	2,200,992	44	0	7	9694
(80,72)	2	2	1	71,138	2,202,714	35	1	1	8108
(41,43)	1	5	0	121,412	2,147,963	40	1	13	9552
(49,66)	1	2	1	50,593	1,996,950	28	0	17	10,340
(77,71)	2	1	1	158,667	917,677	51	0	0	8889
(74,69)	1	3	0	67,066	522,317	36	1	1	4833

**Table 7:** Description of family parameters.

Variable	Range of Values
Household	
Urban	Values are 0, based on the majority of Urban in Table 1
Income	Values range from 20,000–75,000 in increments of 5000 (in Chinese yuan)
Asset	The average value of Asset in the case of Urban = 0 and Gender = 1 in Table 1 is 351,980 (in Chinese yuan)
Edu.	When Income is in the range of low-income households in Table 4 (3-5), household investments are determined based on the regression results in (3-5); when it is in the range of middle-income households in Table 4 (3-6), household investments are determined based on the regression results in (3-6) (in Chinese yuan)
Head of household	
Gender	Values are 1, based on the majority of Gender in Table 1
Age	The average value of Age in the case of Urban = 0 and Gender = 1 in Table 1 is 54
Eduy	The average value of Eduy in the case of Urban = 0 and Gender = 1 in Table 1 is 7
Family members	
Num. of older	The values are 0 and 1
Num. of youth	The number of children in 95% of the families in Table 1 is the upper limit, and the value increases, from 1 to 3
Num. of adults	The values are 2

## Data Availability

The survey used data from the China Family Panel Studies (CFPS 2018) from the Institute of Social Science Survey at Peking University (http://www.isss.pku.edu.cn/cfps/ (accessed on 1 July 2021)).

## Abbreviations

China Family Panel Studies	CFPS
China Social Science Survey Center	ISS
Multi-Agent System	MAS

## References

[1] Ismail Z., Ahmad W.I.W., Hamjah S.H., Astina I.K. (2021). The impact of population ageing: A review. Iran. J. Public Health.

[2] Jedwab R., Loungani P., Yezer A. (2021). Comparing cities in developed and developing countries: Population, land area, building height and crowding. Reg. Sci. Urban Econ..

[3] Jiang Q., Li S., Feldman M.W. (2013). China’s population policy at the crossroads: Social impacts and prospects. Asian J. Soc. Sci..

[4] Xi J. (2022). Hold High the Great Banner of Socialism with Chinese Characteristics and Strive in Unity to Build a Modern Socialist Country in All Respects—Report to the 20th National Congress of the Communist Party of China.

[5] Luo Y., Qi M., Huntsinger C.S., Zhang Q., Xuan X., Wang Y. (2020). Grandparent involvement and preschoolers’ social adjustment in Chinese three-generation families: Examining moderating and mediating effects. Child. Youth Serv. Rev..

[6] Bogan V.L. (2015). Household asset allocation, offspring education, and the sandwich generation. Ecol. Res..

[7] Tao D., Zhang K. (2015). Population aging, intergenerational conflict and public expenditure on education. Educ. Econ..

[8] Silverstein M., Tur-Sinai A., Lewin-Epstein N. (2020). Intergenerational support of older adults by the “mature” sandwich generation: The relevance of national policy regimes. Theor. Inq. Law.

[9] Xiao S. (2016). Intimate power: The intergenerational cooperation and conflicts in childrearing among urban families in contemporary China. J. Chin. Sociol..

[10] Naumann E., Hess M., Steinkopf L. (2015). Die Alterung der Gesellschaft und der Generationenkonflikt in Europa/Aging societies and intergenerational conflict in Europe. Z. Für Soziol..

[11] Bruni M.L., Mammi I., Ugolini C. (2014). Does the extension of primary care practice opening hours reduce the use of emergency services?. J. Health Econ..

[12] Cheng L., Zeng Y., Lu J., Lei X., Shi X. (2022). Long-term care demand and supply among the rural elderly in China: Evidence from CLHLS data. Trends and Determinant Healthy Aging in China.

[13] Luo Y., Su B., Zheng X. (2021). Trends and challenges for population and health during population aging—China, 2015–2050. China CDC Wkly..

[14] Li H. (2021). Population aging, health care burden, and micro-human capital investment. Stat. Decis..

[15] Naoi M., Akabayashi H., Nakamura R., Nozaki K., Sano S., Senoh W., Shikishima C. (2021). Causal effects of family income on educational investment and child outcomes: Evidence from a policy reform in Japan. J. Jpn. Int. Econ..

[16] Dahl G.B., Lochner L. (2012). The impact of family income on child achievement: Evidence from the earned income tax credit. Am. Econ. Rev..

[17] Li Y. (2019). Aging population, child support burden and family human capital investment. J. Xi’an Jiaotong Univ..

[18] Liu N., Qu N., Wang N., Chang N. (2020). Does population aging hinder the accumulation of human capital? Evidence from China. Front. Econ. China.

[19] Li H., Zhang Z., Yang X. (2019). Does the health shock of elderly crowd out household education expenditure?—Mediation effect test based on medical expenditure. Educ. Econ..

[20] Becker G.S. (1960). An economic analysis of fertility, demographic and economic change in developed countries: A conference of the universities. Natl. Bur. Comm. Econ. Res..

[21] Chen Q. (2017). Relaxed population policy, family size and parental investments in children’s education in rural North Western China. Int. J. Educ. Dev..

[22] Kugler A.D., Kumar S. (2017). Preference for Boys, Family Size, and Educational Attainment in India. Demography.

[23] Smith D.S. (1996). ‘The number and quality of children’: Education and marital fertility in early twentieth-century Iowa. J. Soc. Hist..

[24] Hong X., Jiang Y., Luo L., Li H. (2022). The Impact of Two-Child Policy on Early Education and Development in China. Early Educ. Dev..

[25] Bae D., Wickrama K.A.S. (2015). Family socioeconomic status and academic achievement among Korean adolescents: Linking mechanisms of family processes and adolescents’ time use. J. Early Adolesc..

[26] Løken K.V. (2010). Family Income and Children’s Education: Using the Norwegian Oil Boom as a Natural Experiment. Labour Econ..

[27] Fioroni T. (2017). Human capital and fertility: Child vs adult survival. Econ. Bull..

[28] Deng X., Luo X., Wu Y. (2016). The mediating effect of parental involvement between family socioeconomic status and academic performance: Meta-analysis structural equation modeling. Adv. Psychol. Sci..

[29] Lee S.M., Kushner J., Cho S.H. (2007). Effects of Parent’s gender, Child’s gender, and parental involvement on the academic achievement of adolescents in single parent families. Sex Roles.

[30] Quadlin N. (2019). Sibling achievement, sibling gender, and beliefs about parental investment: Evidence from a national survey experiment. Soc. Forces.

[31] Chojnicki X., Rabesandratana P.E. (2018). Aging, human capital, and productivity in France: A generational accounting perspective. Rev. Income Wealth.

[32] Liu B., Yang Z. (2021). Population aging shock and fiscal sustainability in China: Mechanism analysis and effect simulation. Singapore Econ. Rev..

[33] Bucci A., Prettner K. (2020). Endogenous education and the reversal in the relationship between fertility and economic growth. J. Popul. Econ..

[34] Liu D., Xi J., Hall B.J., Fu M., Zhang B., Guo J., Feng X. (2020). Attitudes toward Aging, Social Support and Depression among Older Adults: Difference by Urban and Rural Areas in China. J. Affect. Disord..

[35] Lowenstein A. (1999). Intergenerational Family Relations and Social Support. Z. Gerontol. Geriatr..

[36] Li F. (2016). Physical activity and health in the presence of China’s economic growth: Meeting the public health challenges of the aging population. Sports Med. J. Sport Health Sci..

[37] Leopold T., Raab M. (2013). The temporal structure of intergenerational exchange: A within-family analysis of parent-child reciprocity. J. Aging Stud..

[38] Silverstein M., Conroy S.J., Gans D. (2012). Beyond solidarity, reciprocity and altruism: Moral capital as a unifying concept in intergenerational support for older people. Ageing Soc..

[39] Prinzen K. (2014). Intergenerational ambivalence: New perspectives on intergenerational relationships in the German welfare state. Ageing Soc..

[40] Liu Y. (2015). Motivations of intergenerational transfers: Altruism or exchange—Evidence from Charls. Econ. Theor. Bus. Manag..

[41] Boardman J.D., Alexander K.B., Miech R.A., Macmillan R., Shanahan M.J. (2012). The association between parent’s health and the educational attainment of their children. Soc. Sci. Med..

[42] Yang J., Qiu M. (2016). The impact of education on income inequality and intergenerational mobility. China Econ. Rev..

[43] Mattis J.S., Hammond W.P., Grayman N., Bonacci M., Brennan W., Cowie S.A., Ladyzhenskaya L., So S. (2009). The social production of altruism: Motivations for caring action in a low-income urban community. Am. J. Community Psychol..

[44] Shi J. (2017). The evolvement of family intergenerational relationship in transition: Mechanism, logic, and tension. J. Chin. Sociol..

[45] Yang D., Wen Y. (2013). Egoistic motive, altruistic preferences and inclusive growth: Based on the over lapping generation models with “paternalistic altruism”. Zhejiang Soc. Sci..

[46] Fingerman K.L., Cheng Y.P., Birditt K., Zarit S. (2012). Only as happy as the least happy child: Multiple grown children’s problems and successes and middle-aged parents’ well-being. J. Gerontol. B Psychol. Sci. Soc. Sci..

[47] Yang J., Zheng Y. (2019). Links between perceptions of successes, problems and health outcomes among adult Chinese children: The mediating role of perceptions of parents’ feelings and intergenerational relationships. Front. Psychol..

[48] Tian W., Liu J., Wang Z. (2020). Empirical analysis of relationship between farmers’ income structure and consumption in Guizhou Province in the context of large-scale poverty alleviation. Asian Agric. Res..

[49] Bayar A., İlhan B.Y. (2016). Determinants of household education expenditures: Do poor spend less on education?. Top. Middle East. N. Afr. Econ..

[50] Wang L., Ji Y., Wang Y. (2019). Housing wealth, family income and educational expenditure: A case study of Tianjin. Urban Dev. Stud..

[51] Verba D., Kudinova A. (2019). Absolute value and diversity of household spending: Analysis on International Comparison Program (ICP) 2011 data. Equilibrium. Q. J. Econ. Econ. Policy.

